# Epidemiological Surveillance of Hypodermosis in Cattle from Romania

**DOI:** 10.3390/pathogens12091077

**Published:** 2023-08-24

**Authors:** Gheorghe Dărăbuș, Vasile Daniel Tomoioagă, Tiana Florea, Mirela Imre, Ion Oprescu, Sorin Morariu, Narcisa Mederle, Marius Stelian Ilie

**Affiliations:** Department of Parasitology and Dermatology, University of Life Sciences King Mihai I of Romania Timisoara, Calea Aradului 119, 300645 Timisoara, Romania; gheorghedarabus@usvt.ro (G.D.); tomoioagadaniel90@yahoo.com (V.D.T.); mirela.imre@usvt.ro (M.I.); ionoprescu@usvt.ro (I.O.); sorinmorariu@usvt.ro (S.M.); narcisamederle@usvt.ro (N.M.); mariusilie@usvt.ro (M.S.I.)

**Keywords:** *H. bovis*, cattle, descriptive epidemiology, molecular epidemiology, Romania

## Abstract

Hypodermosis, or warble fly, is an endemic parasitic disease, common in countries from the northern hemisphere. The use of effective insecticides has decreased the frequency of this parasitic disease, with untreated cattle remaining to act as reservoirs. This study focused on assessing the status of hypodermosis in northwestern Romania by means of clinical examination (skin inspection and palpation performed in order to identify nodules) conducted on a number of 11.741 cattle. The study was carried out from March until June 2021. The identified larvae were subject to molecular assays for species identification and genotyping, followed by comparison with data available in the GenBank database. The average prevalence rate of parasitism caused by *Hypoderma* spp. was 0.31%, with values ranging from a minimum of 0.11% to a maximum of 1.32%. The dominant age group among positive animals was the 1–3 years old category and in terms of breed distribution, most positive cases were seen in cows belonging to an indigenous breed: Bruna de Maramures. April was the most prolific month in terms of nodule count/animal. The species identified in our study by means of molecular assays was *H. bovis* with two haplotypes: HB3 and HB8.

## 1. Introduction

Parasitic diseases are cosmopolite health issues, observed in any livestock farming area. The multitude of ectoparasitic species poses a serious threat to effective and productive livestock management due to the negative impact on animal health and productivity [1]. One of these parasitic infestations, especially in dairy cows from temperate areas, is hypodermosis.

The *Hypoderma* genus belongs to the *Oestridae* family which includes approximately 150 species of dipteran flies, their larvae being obligate parasites that develop in the tissues or organs of domestic and wild animals producing miasis. The *Hypoderma* genus includes seven dipteran species according to some authors [2] and six species according to others [3], of which three cause bovine hypodermosis, namely *Hypoderma bovis*, *H. lineatum,* and *H. sinense*. *Hypoderma lineatum* and *Hypoderma bovis* are the most common species.

Hypoderma parasites are encountered solely in the northern hemisphere. Diptera of the genus Hypoderma do not reproduce in Arctic, sub-Arctic, or tropical areas. The presence of cattle suffering from hypodermosis in the southern hemisphere has not resulted in the perpetuation of the species or the survival of the parasite population. The life cycle of warble flies is adapted to seasonal climatic changes in temperate areas. Additionally, warble flies are very well adapted, displaying a wide variability in their life cycle. In Europe, after fertilisation, *H. bovis* females fly and lay eggs on the hairs of cattle from May to September, while *H. lineatum* females fly from May to June. In Spain, the flight of females is encountered as early as April [4]. Adult Hypoderma parasites do not feed, living on reserves from their larval and summer life. During favourable periods (sunny weather), the warble flies only survive for 3–5 days, a period during which they can lay up to 800 eggs [5]. If the weather is cold, unfertilised females can live up to 28 days. *Hypoderma bovis* larvae hatch on about the fourth day after egg-laying and travel through the skin and subcutaneous connective tissue by means of undulating movements along the spinal nerves to the spinal canal. First-stage larvae of *H. lineatum* and *H. sinense* hatch and then migrate over several months through the somatic tissues of animals via the oesophagus [3]. Migration lasts for several months. Subcutaneous settlement in the dorsal and/or lumbar regions (less frequently in other regions) occurs in late winter and spring. First-stage larvae develop into second-stage larvae and then finally into third-stage larvae. This last stage, after slight dehydration, emerges through the skin orifice, falls to the ground, and pupates. Due to the very rich adipose layer formed during the larval stage, females can be fertilized within hours of hatching and lay eggs without feeding [6]. Eggs laid by females are ovoid in shape and have transverse striations, which can be used in differential diagnosis from eggs laid on hairs by lice, which have small alveoli on the eggshell. 

In practical conditions, veterinarians cannot provide an early diagnosis and can only confirm the disease when skin nodules appear. However, immunological diagnosis can be used in the period from December to April, with maximum applicability from December to January. Passive haemagglutination and immunoelectrophoresis methods, like ELISA, can be employed. The antigen used is a protease (hypodermin C) secreted by first-stage larvae during migration. ELISA, having a very good specificity, is suitable for mass diagnosis because it can be performed on serum and whey samples [7]. 

The characteristics of bovine hypodermosis include chronicity, seasonality, and benignancy, with the main clinical signs consisting of subcutaneous nodules emerging in the dorso-lumbar region. The signs of ectoparasitic infections can be observed by the naked eye and do not require special equipment or diagnostic methods. Hair and skin appear affected, and the animals become restless due to intense pruritus.

The two species of warble flies common in Romania, namely *H. bovis* and *H. lineatum,* can be differentiated based on the morphological characteristics of third-stage larvae. Larvae of *H. bovis* have spines on the posterior that end only up to body segments four or five on the dorsal side and six on the ventral side, while the posterior stigmas are funnel shaped. In *H. lineatum*, spines are present up to the fourth body segment on the dorsal face and up to the seventh segment on the ventral face, and the posterior stigmas are flat [6]. 

Numerous studies have dealt with the comparative presentation and description of *H. bovis*, *H. lineatum*, *H. diana*, *H. actaeon*, and *H. tarandi* species, using both electron microscopy and molecular identification based on mitochondrial genes of cytochrome oxidase subunit I [8,9]. The stress caused by these clinical manifestations translates into decreased productivity due to the fact that animals cannot rest, eat or ruminate properly [2], especially if associated with internal parasitosis [10]. Grazing disturbance caused by flies during egg laying, especially in the case of *H. bovis* species, can lead to a behaviour known as gadding. Parasitism is associated with reduced reproductive indices, the possibility of inflicting self-injury, reduced weight gain, and reduced milk production. Even more important losses are related to the migration of *Hypoderma* larvae, which lead to damage of tissues, resulting in the seizure of affected meat in slaughtered cattle, and perforation of the skin, resulting in a reduction in its value [11]. Despite programs aimed to control hypodermosis being developed and implemented in the middle of the 20th century in many European countries, the disease is still present in most areas and causes significant economic losses for farmers facing this threat [10,12]. The study of hypodermosis is also important because the infection can be identified in humans as well. Cases of human infection with *Hypoderma* sp. are rare, especially in Europe. However, in one case report, a farmer who had never travelled outside Italy was presented with an *H. lineatum* infection, confirmed by molecular analysis [13]. Puente et al. (2010) [14] reported an *H. sinense* infection in a citizen who presented with abdominal pain and inflammation of the right inguinal and testicular area after travelling to northern India. The limited reports on the epidemiological situation of hypodermosis in Romania determined our engagement in the process of clarifying the lesser-known aspects regarding the prevalence of this disease and the species/strains of *Hypoderma* involved. 

## 2. Materials and Methods

The study was carried out in five localities from the northwestern part of Romania during March, April, May, and June 2021. 

The total number of studied animals was 11.741, including cattle belonging to two indigenous breeds (Brună de Maramureș and Bălțată Românească), as well as mixed-breed animals.

**Figure 1:**
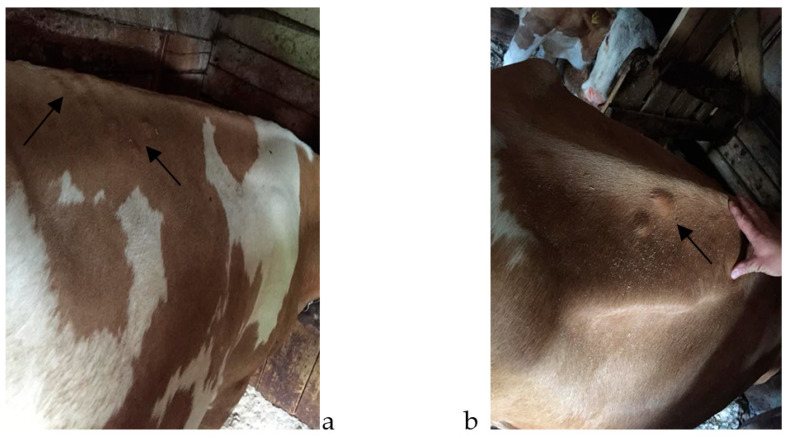
(**a**,**b**)—*Hypoderma* spp. nodules in the dorsal area of indigenous-breed (Bălțată Românească) cattle (see arrows).

**Figure 2:**
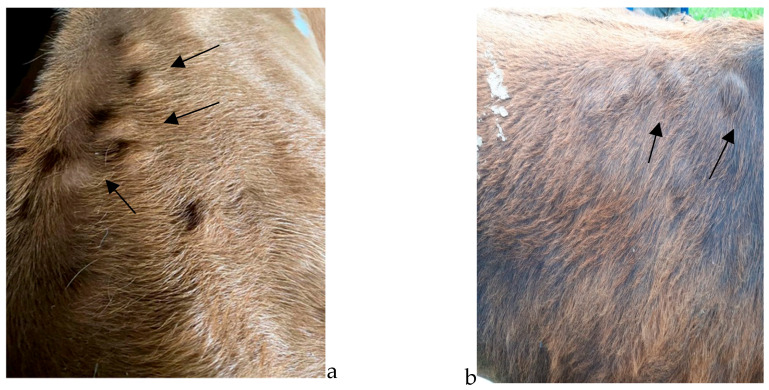
(**a**,**b**)—Multiple *Hypoderma* spp. nodules in the dorsal area of indigenous-breed (Brună de Maramureș) cattle (see arrows).

The nodules (Figure 1 and Figure 2) were identified visually, followed by a palpatory confirmation of their presence on the body of the examined animals. Data regarding age, breed, and monthly numeric evolution of nodules were recorded for the animals found clinically positive for the presence of *Hypoderma* spp. 

Samples consisting of stage three *Hypoderma* spp. larvae were collected from the positive animals and prepared for further evaluation. Thus, they were fixed by immersion in 70% ethanol and examined with the aid of an optic microscope in order to properly observe the morphological characteristics unique to each species. 

Subsequently, molecular analysis was employed as the method for species identification. The DNA extraction step was performed using the ISOLATE II Genomic DNA Kit (BIOLINE^®^), following protocol for tissues, in accordance with the manufacturer’s recommendations.

The PCR reaction was performed according to the technique described by Otranto et al., 2003 [15], with some minor changes. Amplification itself was carried out by classic PCR and was based on amplifying a partial DNA fragment of the COI gene with a size of ~579 bp for *Hypoderma* spp.

The primers used were UEA7, 5′TACAGTTGGAATAGACGTTGATAC-3 and the reverse primer UEA10, 5′ TCCAATGCACTAATCTGCCATATTA-3′ [15]. Amplification was carried out according to the protocol described in the article and modified according to the requirements of the mixture. MyTaqTM Red Mix (BIOLINE^®^) was used to carry out the reaction. The final volume of the PCR reaction was 25 µL.

The amplification program was carried out with the thermocycler My Cycler (BioRad^®^, Berkeley, CA, USA). This program included DNA denaturation steps at 95 °C for 1 min; 32 denaturation cycles at 95 °C, time 30 s, hybridization at 60 °C, time 30 s and extension at 72 °C, time 30 s; and incubation at 4 °C.

The analysis and control of the amplicons were carried out by horizontal electrophoresis in a system submerged in 1.5% agarose gel, with the addition of the fluorescent dye MidoriGreen (Nippon Genetics^®^ Europe).

The 100 bp DNA Ladder (Bioline) marker was used in the first well of the gel.

For species confirmation and genotyping, PCR products were sequenced and compared with those available in the GenBank database using BLASTn alignment (http://blast.NCBI.NLM.nih.gov/blast.cgi, accessed on 24 July 2021).

## 3. Results

### 3.1. Descriptive Epidemiology

**Figure 3:**
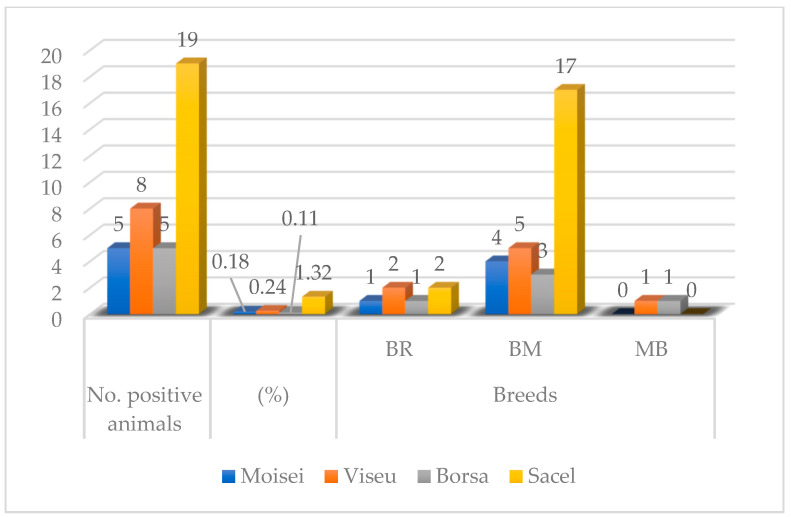
Graphical representation of positive cases according to the sampling location. BR = Bălțată Românescă; BM—Brună de Maramureș; MB—Mixed Breed.

**Figure 4:**
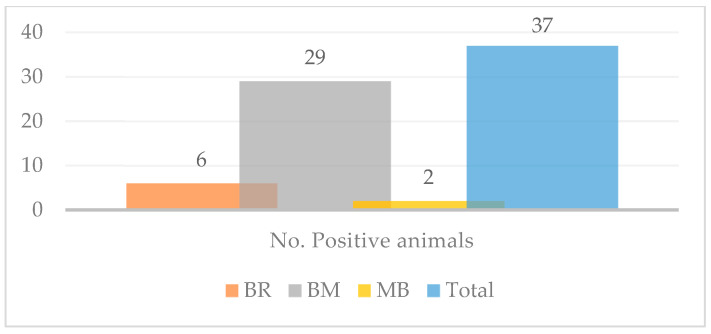
Distribution of positive cases according to breed. BR = Bălțată Românescă; BM—Brună de Maramureș; MB—Mixed Breed.

The overall prevalence rate that resulted from our study was 0.31% (*n =* 21), with values ranging from 0.11% (lowest point) to 1.32% (highest point) (Table 1, Figure 3 and Figure 4). 

**Figure 5:**
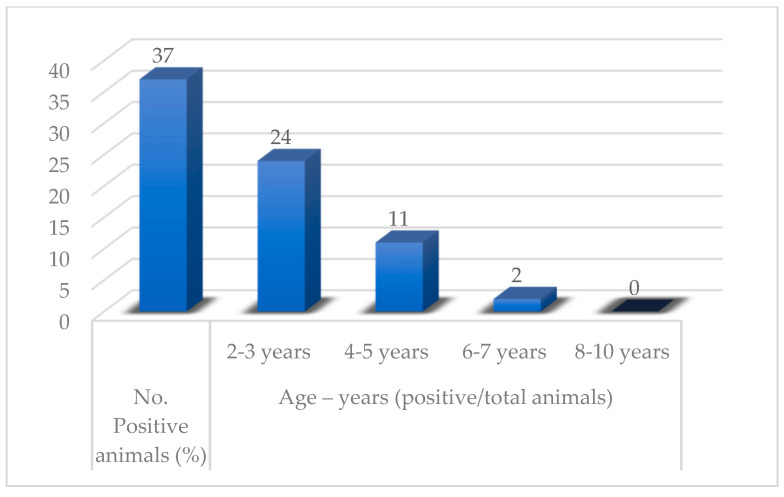
Distribution of positive cases according to age.

The age range of the positive animals was 2–6 years; however, a higher percentage of positive cases was recorded in animals aged 1–3 years (Table 2, Figure 5).

Positive cases originated from isolated geographic regions, with traditional farming methods and where previous prevention treatments were not properly implemented.

**Figure 6:**
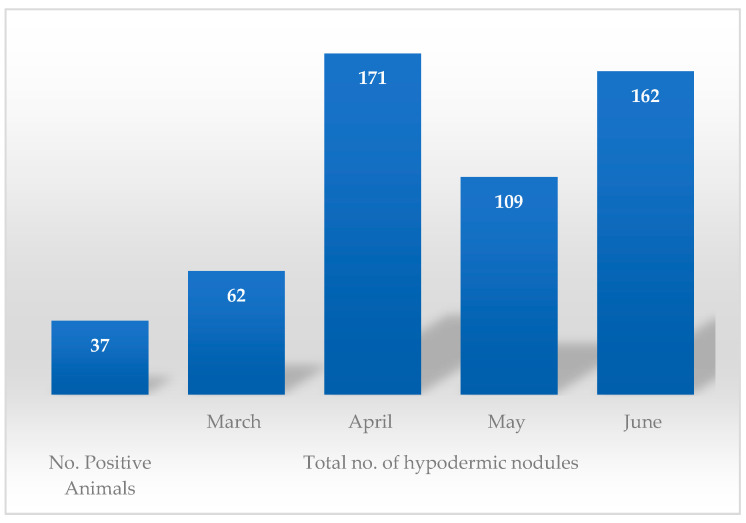
Monthly variation of *Hypoderma* nodule count.

The very first positive cases were discovered in March however, the most prolific month in terms of case positivity and nodule count (*n* = 15, the maximum number of nodules counted on an individual) was April (Table 3, Figure 6). The animals were not examined concomitantly (some of them were examined in March, some in April, and others in May and June); thus, there is no way of telling whether the nodules found on the animals examined during April or May, for example, had been there prior to the examination or not. Certain breed predispositions (Table 4 and Table 5, Figure 4) proved statistically significant, namely the predisposition of Brună de Maramureș cattle compared with half-breeds (*p* < 0.001). 

### 3.2. Molecular Epidemiology

All five samples were classified as *H. bovis* by molecular identification of *Hypoderma* spp. larvae 3 (performed by PCR). Following the BLASTn analysis of the sequences obtained from the amplification of the extracted DNA, it was observed that the identified sequences were identical to the isolates: GU984815.1, GU984814.1, KT600279.1. Based on mt-CO1 gene sequence analysis, haplotype HB3 and haplotype HB8 were identified. The results obtained by PCR confirm the results of the morphological identification of the *H. bovis* species. 

## 4. Discussion

Prevalence rates for warbles vary widely according to region, and the rates have seen a significant drop in numbers in the past years, even up to eradication in some countries such as the UK, where the prevalence rate prior to eradication was reported around 40% in a population of 10 million cattle [15]. The prevalence rate of parasitism with *H. bovis* resulting from this study is fairly low compared with previously conducted studies, which reported prevalence rates between 32 and 43%, [16,17], 16.21% in Southern Romania [18] or with other countries: up to 85% in Italy [19,20], 67% in Turkey, 23% in Iraq [21], 43% in Belgium, 40% in France, 37.4% in Greece [22], 52.3% in Spain, 16% in Poland, 20% in Switzerland, up to 80% in China [23], and 31.1% in Albania [24]. The prevalence rate recorded during our study was 0.17%. This low rate may be due to easier access and, consequently, more frequent application of control measures (prevention treatments with insect repellents and seasonal treatments with Ivermectin for the treatment and prevention of other internal parasitic diseases). The results of the study show a significant decrease in rates recorded between 2005 (32–43%) [16,17] and 2023 (0.31%), which might outline the possibility of an emerging eradication of this disease. The difference in rates between our study conducted in the northern part of the country and the one conducted by Gorcea et al. [18] in the southern part may also be explained by high differences in temperatures between the two areas, conditions being clearly more favourable for the development of warble flies in the southern part of Romania. 

At the end of the 20th century, a hypodermosis control programme was implemented in many European countries which significantly reduced the prevalence of hypodermosis [12]. However, in Poland, after the end of the programme, the prevalence again reached 20% up to 100% in cattle herds [25]. The fact that adults can fly up to 5 km in distance contributes to the spread of this parasite and to the relative importance of parasitological control measures in a given region [26]. In terms of age predisposition, the results of this study are similar to other reports that reveal a higher rate of infection among young animals (over the age of 1 year) [27] compared with older individuals, which develop immunity against warble larvae with ageing. Therefore, age can be a risk factor. Thus, young animals under 1 year of age, being usually kept in shelters and generally not allowed to graze freely, have a lower prevalence [24]. In contrast, animals aged 1 to 3 years are highly susceptible to both *H. bovis* and *H. lineatum*. Adult cattle may have partial or even total resistance. Jersey and Holstein breeds are very susceptible to *H. bovis* parasitism, while the Australian Sahival breed is resistant. Vitamin A deficiency may be a risk factor facilitating skin penetration by larvae I, which has been demonstrated for *H. lineatum* [16].

The increase in the number of nodules in April, May, and June is probably due to the period when the eggs were laid, i.e., the period when the females carried out their oviposition flight in the previous year. The oviposition periods can be influenced by many factors: the biology of the parasite, species of Hypoderma, climatic factors, age and species of the parasitized animals, possible treatments, insecticides used, etc. Species differentiation is an important tool when it comes to prevention, especially considering the behavioural variations among *Hypoderma* spp. (for example, *H. bovis* and *H. lineatum* differ in terms of animal approach, egg laying and size, migration pattern of larvae inside the affected host, pathogeny, severity of lesions, etc.). According to data presented by Hassan et al. [15], the most common species of *Hypoderma* are *H. bovis* and *H. lineatum* when it comes to domestic cattle. *H. bovis* is frequently reported in countries from the northern hemisphere, and its pathogeny is of greater importance to farmers, considering the severity of lesions that this infection might induce if treatment is not instituted promptly (when first-stage larvae are in the peri-rachidian channel, in the absence of treatment, they might induce paralysis of the hind quarters) [13]. The results of the morphological identification doubled by PCR assays based on mitochondrial COI sequencing for the identification of *H. bovis* [28] performed during our study revealed the presence of *H. bovis* with two haplotypes: HB3 and HB8. Another study based on molecular characterisation, conducted by Otranto et al. [29], revealed similar results. Other authors [30] have reported the presence of *H. diana* in deer found dead in forests in Romania. 

## 5. Conclusions

The prevalence in northwestern Romania was 0.31%, with variations among the four studied areas ranging from 0.11% to a maximum rate of 1.32%. Warbles is a disease affecting mostly young individuals, especially animals from the 2–3 (*p* < 0.001) years of age category, while in terms of breed, statistical significance (*p* < 0.001) was noted between half breeds and Brună de Maramureș. The highest *Hypoderma* nodule count was noted in April. PCR assays and morphological identification methods revealed the presence of the *H. bovis* species, with two haplotypes: HB3 and HB8. For a more complete understanding of the epidemiological situation of this disease, we recommend future studies be conducted in other geographical parts of our country in order to update the status of this apparently disappearing disease in cattle from Romania. 

## Figures and Tables

**Table 1:** Prevalence of parasitism with *H. bovis* in Northwestern Romania.

Location	No. of Examined Animals	No. of Positive Animals (%)	Breeds		
			BR	BM	MB
Moisei	2667	5 (0.18)	1	4	0
Vișeu	3215	8 (0.24)	2	5	1
Borșa	4420	5 (0.11)	1	3	1
Săcel	1439	19(1.32)	2	17	0
TOTAL	11,741	37 (0.31)	6	29	2

BR = Bălțată Românescă; BM—Brună de Maramureș; MB—Mixed Breed.

**Table 2:** Prevalence of parasitism with *H. bovis* according to the age group.

Breed	No. of Examined Animals	No. of Positive Animals (%)	Age Group (Positive/Total No. of Animals)			
			2–3 Years	4–5 Years	6–7 Years	8–10 Years
BR	2843	6 (0.21)	5/379	0/793	1/632	0/1039
BM	6320	29 (0.45)	18/847	10/1760	1/1108	0/2605
MB	2578	2 (0.07)	1/322	1/637	0/701	0/918
TOTAL	11,741	37 (0.31)	24/1548	11/3190	2/2441	0/4562

**Table 3:** Identification of hypodermic nodules according to month.

Location	No. of Positive Animals	Total No. of Hypodermic Nodules			
		March (Max–Min)	April (Max–Min)	May (Max–Min)	June (Max–Min)
Moisei	5	17 (2–5)	30 (4–8)	19 (1–6)	11 (1–4)
Vișeu	8	21 (1–5)	70 (4–15)	47 (2–12)	25 (2–7)
Borșa	5	15 (2–4)	48 (6–14)	25 (3–7)	20 (1–5)
Săcel	19	9 (2–4)	23 (5–10)	18 (4–10)	106 (1–11)
Total	37	62 (1–5)	171 (4–15)	109 (1–12)	162 (1–11)

**Table 4:** Statistical significance of comparisons between age groups and breeds.

**AGE**			
2–3	vs.	4–5	0.0001
2–3		6–7	0.0001
2–3		8–10	0.0001
4–5		6–7	0.0500
4–5		8–10	0.0001
6–7		8–10	0.1216
**BREED**			
BR		BM	0.0979
BR		HB	0.2935
BM		HB	0.0044

**Table 5:** Statistical significance of comparisons between monthly nodule counts in the studied cases.

March (Max–Min)	April (Max–Min)	*p* value equals 0.0450 By conventional criteria, this difference is considered to be statistically significant.
17 (2–5)	30 (4–8)	
21 (1–5)	70 (4–15)	
15 (2–4)	48 (6–14)	
9 (2–4)	23 (5–10)	
March (Max–Min)	May (Max–Min)	*p* value equals 0.1543 By conventional criteria, this difference is considered to be not statistically significant.
17 (2–5)	19 (1–6)	
21 (1–5)	47 (2–12)	
15 (2–4)	25 (3–7)	
9 (2–4)	18 (4–10)	
March (Max–Min)	June (Max–Min)	*p* value equals 0.3025 By conventional criteria, this difference is considered to be not statistically significant.
17 (2–5)	11 (1–4)	
21 (1–5)	25 (2–7)	
15 (2–4)	20 (1–5)	
9 (2–4)	106 (1–11)	
April (Max–Min)	May (Max–Min)	*p* value equals 0.2609 By conventional criteria, this difference is considered to be not statistically significant.
30 (4–8)	19 (1–6)	
70 (4–15)	47 (2–12)	
48 (6–14)	25 (3–7)	
23 (5–10)	18 (4–10)	
April (Max–Min)	June (Max–Min)	*p* value equals 0.9295 By conventional criteria, this difference is considered to be not statistically significant.
30 (4–8)	11 (1–4)	
70 (4–15)	25 (2–7)	
48 (6–14)	20 (1–5)	
23 (5–10)	106 (1–11)	
May (Max–Min)	June (Max–Min)	*p* value equals 0.5861 By conventional criteria, this difference is considered to be not statistically significant.
19 (1–6)	11 (1–4)	
47 (2–12)	25 (2–7)	
25 (3–7)	20 (1–5)	
18 (4–10)	106 (1–11)	
